# Korean Version of Child Perceptions Questionnaire and Dental Caries among Korean Children

**DOI:** 10.1371/journal.pone.0116011

**Published:** 2015-02-12

**Authors:** Hye-Sun Shin, Dong-Hun Han, Myung-Seop Shin, Hyun-Jin Lee, Mi-Sun Kim, Hyun-Duck Kim

**Affiliations:** 1 Department of Preventive and Social Dentistry, School of Dentistry, Seoul National University, Seoul, Korea; 2 Dental Research Institute, School of Dentistry, Seoul National University, Seoul, Korea; TNO, NETHERLANDS

## Abstract

Although dental caries has been a major oral health problem for children, the association between dental caries and oral health related quality of life has been still controversial. This study aims to evaluate the association between the Korean version of the Child Perceptions Questionnaire (K-CPQ) and dental caries among Korean children. Eight hundred one school children aged 8 to 14 years participated in this study. After the K-CPQ was validated we performed an association study. The K-CPQ was self-reported. Dental caries were evaluated by dentists using the World Health Organization Index. Correlation analyses (intraclass correlation coefficient, Cronbach’s alpha and Pearson’s correlation coefficient [r]) and linear regression models (partial r) including age, gender and type of school were applied. Untreated deciduous dental caries was associated with the K-CPQ_8-10_ overall score (partial r = 0.15, *P* <0.05). The link was highlighted in the domains of functional limitation and emotional well-being. Filled teeth due to caries (FT) was associated with the K-CPQ_11-14_ overall domain (partial r = 0.14, *P* = 0.002) as well as with the oral symptoms domain (partial r = 0.16, *P* = 0.001). This association was highlighted among public school children. Our data indicate that K-CPQ was independently associated with dental caries. The K-CPQ could be a practical tool to evaluate the subjective oral health among Korean children aged 8 to 14.

## Introduction

Measuring oral health-related quality of life (OHRQOL) provides essential information when assessing the treatment needs of individuals and populations, making clinical decisions and evaluating interventions, services and programs [1]. Hence, OHRQOL has emerged as an important indicator of oral health [2]. In dentistry, most of OHRQOL tools have been used for adult populations [1,3–8]. However, the use of OHRQOL measures for children has lagged behind its use for adults. The OHRQOL instruments designed to assess the impact of oral conditions on the daily lives of children and adolescents are the Child- Oral Impacts on Daily Performances (Child-OIDP) [9], the Early Childhood Oral Health Impact Scale (ECOHIS) [10], and the Child Perceptions Questionnaire (CPQ) [11,12].

Two instruments of CPQ were developed to measure the OHRQOL among English speaking children aged 8–10 years [11,12] and 11–14 years [11,12] at the University of Toronto. It includes four domains of oral symptoms: oral symptoms (OS), functional limitations (FL), emotional well-being (EW) and social well-being (SW), conceptually. Especially, the validity of CPQ_8–10_ has been demonstrated in English-speaking children in Canada [11] and Australia [13]. The translated and modified versions of CPQ_8–10_ have been also developed in Denmark [14], Brazil [15–17] and Mexico [18]. The CPQ_11–14_ been has been tested in Canada, [11] the United Kingdom [19], New Zealand [20], Australia [13] and translated and modified in China [21], Brazil [15], Nigeria [22], Denmark [14], Thailand [23], Germany [24], Saudi Arabia [25], Italy [26], and Sweden [27].

The CPQ_8–10_ has been previously developed and tested conventionally in clinical samples of children in Canada [12] and Denmark [14]. The clinical groups were targeted to the children with dental caries and clefts of the lip and/or the palate. The CPQ_11–14_ has been largely evaluated among convenient samples of patients with specific diseases or disorders such as cleft lip/palate or malocclusion [11], temporomandibular disorder [28], wearing fixed orthodontic appliances [29], developmental enamel defects [30], or traumatic dental injury [31]. Since dental caries is the major oral health problem for children, the evaluation of the relationship between dental caries and OHRQOL is especially important among children. The association between the caries experience and CPQ has been still controversial: for CPQ_8–10_, a positive overall-association in Canadian clinical participants [12], and Mexican children was found [32], no overall-association has been reported in Australian children [13] and Brazilian school children [15]. For CPQ_11–14_, out of seven reports among the general population, a New Zealand study [20] and an Australian study [13] both reported a positive association of caries in children with the CPQ_11–14_. However, other studies conducted in Germany [24], Brazil [15], Thailand [23], Sweden [27], and Nigeria [22] reported no associations.

Moreover, the association between dental caries and CPQ has not been reported in Korea. Hence, this study aimed to evaluate the association between CPQ and dental caries among Korean school children aged 8 to 14 years. To meet the main aim of this study, the Korean version of CPQ (K-CPQ) was developed using forward-backward translation and validated with overall subjective oral health status prior to the main study.

## Materials and Methods

### Ethic statement

This study was approved by the Institutional Review Board for Human Subjects of the School of Dentistry, Seoul National University (approval number: S—020060000) and the Pusan National University Hospital (approval number: 2009016). All children participated voluntarily and provided written informed consent from their parents or guardians.

### Study design

A cross-sectional study was designed and performed to test the hypothesis that CPQ is associated with dental caries.

Under the condition of α (Type I) error of 0.05, β (Type II) error of 0.2 and Pearson’s correlation coefficient (r) of 0.15 between dental caries and the K-CPQ_8–10_ from the pilot study of 39 children, a total of 346 participants were estimated to be needed for K-CPQ_8–10_ association study. Under the condition of Pearson’s r of 0.14 between dental caries and the K-CPQ_11–14_ from the pilot study of 47 children, a total of 371 study participants were estimated to be needed for K-CPQ_11–14_ association study.

We recruited school children using three-stage stratified cluster sampling method. First stratum was two-region: metropolitan cities and general cities which cover around 90% of Korean population. Next stratum was two-type of schools: national and public primary schools, which address over 95% of Korean primary schools. Public schools are run by the local government such as cities or counties. National schools are run by the central government as primary education models. The final stratum was the school cluster. We selected four schools under the agreement of the school authority. All of the 1st to 6th graders registered in the final school clusters were volunteer participants in this study under the permission from their parents or guardians.

Total target children were 412 K-CPQ_8–10_ and 504 K-CPQ_11–14_: one public school and one national school in Seoul (the largest metropolitan city), two public schools in Busan (the second largest metropolitan city), and one public school in Ansan (a rural city in GyungGi province). The participants completed self-reported questionnaires about K-CPQ and were examined caries experience by two dentists. Of the 916 children initially selected, 115 children had missing values in their dental examination or their questionnaires and 801 children (355 children for K-CPQ_8–10_ and 446 children for K-CPQ_11–14_) were remained in the final analysis (Table 1).

**Table 1:** Characteristics of participants.

Variables	CPQ_8–10_		CPQ_11–14_	
	n	%	n	%
Total	355	100	446	100
Gender				
Boy	166	46.8	219	49.1
Girl	189	53.2	227	50.9
Age				
8 years (11 years)	60	16.9	(114)	(25.6)
9 years (12 years)	53	14.9	(52)	(11.6)
10 years (13 years)	242	68.2	(259)	(58.1)
(14 years)			(21)	(4.7)
School				
Rural public school	39	11.0	47	10.5
Urban public school	198	55.8	260	58.3
Urban national school	118	33.2	139	31.2

### Construction of K-CPQ

Three Korean dentists (Drs. HD Kim, DH Han, and CS Kim) who were fluent in both Korean and English participated in the forward-backward translation of the original version of CPQ. Two Korean dentists (Drs. DH Han and CS Kim) performed a forward translation. The backward translation from the Korean version into the new English version was performed by the other Korean dentist (Dr. HD Kim) without knowing the original English version. This re-translated English version was compared with the original English version. Two forward-translators and one backward-translator discussed about the divergences that were found. Together with the solved discrepancies to consider cross-cultural equivalence, K-CPQs were finalized.

### Validation of K-CPQ

One-week interval test-retest reliability of the questionnaires from the children participated was assessed by intraclass correlation coefficients (ICCs) of K-CPQ overall and domain scores among K-CPQ_8–10_ group (n = 105) and K-CPQ_11–14_ group (n = 114), respectively (Table 2). Internal consistency among questionnaires of K-CPQ was also measured by Cronbach’s alpha.

**Table 2:** Intraclass correlation coefficient (ICCs) for test-retest reliability and Cronbach’ α for internal consistency of the Korean version of Child Perceptions Questionnaire.

CPQ Scale	CPQ_8–10_			CPQ_11–14_		
	Item Number	ICC (95% CI ^a^) (n = 105)	α^b^ (n = 355)	Item Number	ICC (95% CI ^a^) (n = 114)	α^b^ (n = 446)
Overall	25	0.79 (0.66–0.86)	0.85	37	0.85 (0.78–0.89)	0.89
Oral symptoms	5	0.74 (0.62–0.82)	0.57	6	0.67 (0.53–0.78)	0.64
Functional limitations	5	0.77 (0.65–0.84)	0.70	9	0.88 (0.83–0.92)	0.70
Emotional well-being	5	0.79 (0.68–0.86)	0.77	9	0.88 (0.83–0.92)	0.90
Social well-being	10	0.22(-0.14–0.47)	0.73	13	0.72 (0.60–0.81)	0.79

^a^ CI denotes confidence interval.

^b^ α denotes Cronbach’ α.

For analyzing construct validity, Pearson’s correlation coefficients (r) between the K-CPQ scores and the two global subjective oral health indicators were determined.

### Dental caries data collection

Prior to the main survey, the dental examiners underwent a calibration session for the assessment of dental caries. The first step of the procedure was dictation and discussion using slides to verify the validity in accordance with the criteria of dental caries by the World Health Organization (WHO) criteria [33]. The next step was a test-retest dental examination on 409 teeth from 19 children to verify the reproducibility. After achieving the inter/intra-examiner Kappa value over 0.8, the main survey was performed. During the main survey, 80 participants, 10% of total participants were selected to evaluate the test-retest reliability. The intra-examiner reliability of the two dentists resulted in Kappa values of 0.84 and 0.97 for the total caries experience index: the sum of decayed, missing, filled permanent teeth index (DMFT) and decayed, filled deciduous teeth index (dft) in 41 children 8–10 years old and Kappa values of 0.87 for DFMT and 0.94 for dft in 39 children 11–14 years old. The inter-examiner reliability between two dentists had Kappa values of 0.62 for DMFT and 0.66 for dft. All examinations for dental caries took place under an indirect daylight at the children’s classroom according to the guideline of the WHO.

### K-CPQ data collection

Each child completed the form of age-specific K-CPQ just prior to the dental examination. Lower scores indicate a better OHRQOL.

### K-CPQ_8–10_

The full set for the original version of CPQ_8–10_ has two different instruments: two global questions about subjective dental health and 25 questionnaires for CPQ_8–10_ four subdomains. Each of three domains (oral symptoms, functional limitations and emotional well-being) has five questionnaires equally and the other social well-being domain has ten questionnaires. Hence, the K-CPQ_8–10_ has the same sets as the original English version. For the two global questions on dental health, they were worded as follows: “When you think about your teeth and mouth, would you say that they are?” and “How much do your teeth or mouth bother you in your everyday life?” Response options were a 4-point rating scale which was rated from “very good” to “poor” for the self-reported rating of the subjective oral symptom and a 5-point rating scale which was rated from “not at all” to “very much” for the self-reported assessment of bothering due to oral health. The K-CPQ_8–10_ questionnaires about four domains (oral symptoms, functional limitations, emotional well-being, and social well-being) were about the frequency of events during the previous four weeks. Items of the K-CPQ_8–10_ used a 5-point rating scale with response options of ‘never’ (code = 0), ‘once or twice’ (code = 1), ‘sometimes’ (code = 2), ‘often’ (code = 3), ‘very often’ (code = 4)’. K-CPQ_8–10_ overall and domain scores were calculated by summing up the scores of the responses to the total 25 items and 5–10 items corresponding to the domains, respectively.

### K-CPQ_11–14_

The original version of the CPQ_11–14_ has two separate sections: two global questions regarding subjective dental health and 37 questions distributed among four domains: oral symptoms (6 questionnaires), functional limitations (9 questionnaires), emotional well-being (9 questionnaires) and social well-being (13 questionnaires). The K-CPQ_11–14_ has the same sets of questions as the original English version. In terms of global questions, they were worded as follows: “Would you say the overall health of your teeth, lips, mouth, and jaws is?” and “How much does the condition of your teeth, lips, mouth or jaws affect your life overall?” Response options for the two global questions about dental health were a 5-point rating scale which ranged from “excellent” to “poor” for the subjective oral health question, and ranged from “not at all” to “very much” for the overall well-being question. The K-CPQ_11–14_ questions in the four domains (oral symptoms, functional limitations, emotional well-being, and social well-being) asked about the frequency of these events during the four weeks prior to taking the questionnaire. These K-CPQ_11–14_ questions used a 5-point rating scale and the response options were ‘never’ (code = 0), ‘once or twice’ (code = 1), ‘sometimes’ (code = 2), ‘often’ (code = 3), and ‘very often’ (code = 4)’. The overall K-CPQ_11–14_ score and the domain scores were calculated by summing up the scores of the responses of all 37 items and the 6–13 items corresponding to each of the domains, respectively.

### Confounding data collection

Confounders are composed of age, gender and type of schools. All of the data in relation to the confounders were obtained from the information in the school register. Type of school was included as a surrogate of a socioeconomic factor.

### Statistical analysis

Correlation analyses were applied to evaluate ICCs and Cronbach’s alpha of the K-CPQ overall and domain scores. Pearson’s correlation coefficient (r) between the K-CPQ scores and the two global subjective oral health indicators were determined using correlation analyses.

Associations between K-CPQ overall and domain scores and caries experience index in deciduous and permanent teeth were assessed by using Pearson’s correlation coefficient. Multivariable linear regression models were performed to investigate the partial correlation coefficient (partial r) adjusted for age, gender and the type of schools. Moreover, subgroup analyses by the type of schools were applied to evaluate whether the type of schools induce the effect modification on the association.

## Results

### Participants

The final analysis was conducted among 355 children for K-CPQ_8–10_ and 446 children for K-CPQ_11–14_, respectively. Majorities of participants are 10 and 13 years of age and urban children, however males and females are in the similar proportion (Table 1).

### Validation of Child Perceptions Questionnaire (K-CPQ_8–10_)

The ICC for the K-CPQ_8–10_ overall score was 0.79 and those of domain scores ranged from 0.22 to 0.79 (Table 2). The social well-being domain score had the lowest the ICCs among domain scores. Cronbach’s alpha was 0.85 for K-CPQ_8–10_ overall score and it ranged from 0.57 to 0.77 for K-CPQ_8–10_ domain scores. The oral symptom domain had lower internal consistency reliability than the other domains (0.57 versus 0.70–0.77). The K-CPQ_8–10_ overall scores had positive correlation with subjective oral health (partial *r* = 0.37, *P* < 0.001) and bothering due to oral health (partial *r* = 0.32, *P* < 0.001) (Table 3). Out of all domains, oral symptoms domain had the highest association with both subjective oral symptoms (partial *r* = 0.477, *P* < 0.001) and bothering due to oral health (partial *r* = 0.322, *P* < 0.001).

**Table 3:** Validation of K-CPQ scores with subjective oral health.

CPQ Scale	CPQ_8–10_ (n = 355)				CPQ_11–14_ (n = 446)			
	Subjective Oral symptoms		Bothered by their oral health		Subjective Oral health		Overall well-being	
	r ^a^	partial r ^b^	r ^a^	partial r ^b^	r ^a^	partial r ^b^	r ^a^	partial r ^b^
Overall	**0.353**	**0.366**	**0.314**	**0.323**	**0.307**	**0.318**	**0.176**	**0.193**
Oral symptoms	**0.464**	**0.477**	**0.318**	**0.322**	**0.344**	**0.346**	**0.229**	**0.237**
Functional limitations	**0.210**	**0.215**	**0.242**	**0.253**	**0.248**	**0.269**	**0.121**	**0.148**
Emotional well-being	**0.278**	**0.288**	**0.238**	**0.241**	**0.24**	**0.223**	**0.165**	**0.156**
Social well-being	**0.140**	**0.145**	**0.172**	**0.178**	**0.128**	**0.151**	0.034	0.056

**Bold** type denotes statistical significance at *p*-value <0.05.

^a^ r denotes Pearson’s correlation coefficient.

^b^ partial r denotes partial correlation coefficient adjusted for age, gender and school type by multivariable linear regression model.

### Validation of Child Perceptions Questionnaire (K-CPQ_11–14_)

The ICCs for the K-CPQ_11–14_ overall score was 0.85 and those of the domain scores ranged from 0.67 to 0.88 (Table 2). Among the domain scores, the oral symptoms domain had the lowest ICC value. The Cronbach’s alpha was 0.89 for the K-CPQ_11–14_ overall score and ranged from 0.64 to 0.90 for the K-CPQ_11–14_ domain scores. The oral symptom domain had a lower internal consistency reliability score than the other domains (0.64 versus 0.70–0.90).

The K-CPQ_11–14_ overall scores had a positive correlation with subjective oral health (partial *r* = 0.32, *P* < 0.001) and overall well-being (partial *r* = 0.19, *P* < 0.001) (Table 3). Out of all domains, the oral symptoms domain had the highest association with both subjective oral health (partial *r* = 0.35, *P* < 0.001) and overall well-being (partial *r* = 0.24, *P* < 0.001). However, social well-being had the lowest correlation with overall well-being (partial *r* = 0.056, *P* < 0.239).

### Association between dental caries and K-CPQ_8–10_

The descriptive statistics showed that the K-CPQ_8–10_ overall score ranged from 0 to 48, with the mean of 7.16 and the standard deviation of 7.61. There were nine children (2.5% of total) with floor effect but no children with ceiling effect. When the responses “often” or “very often” are counted as negative dichotomous value, the proportions of those reported negative impacts on oral symptoms, functional limitations, emotional well-being and social well-being were 16.1%, 9.3%, 5.4%, and 7.9%, respectively. In terms of the objective oral health status (Table 4), K-CPQ_8–10_ overall score had positive correlation with the untreated dental caries on deciduous teeth (dt) (partial *r* = 0.15, p = 0.003). The score of dt was associated with only two domain scores: functional limitation (partial *r* = 0.12, p = 0.015) and emotional well-being (partial *r* = 0.23, p < 0.001). Moreover, permanent and deciduous dental caries experience (the sum of dt and decayed permanent teeth index [DT] [dt+DT]) were also positively associated with K-CPQ_8–10_ overall score (partial *r* = 0.13, p = 0.011), functional limitation score (partial r = 0.11, p = 0.035) and emotional well-being domain score (partial r = 0.21, p < 0001). However, DT and DMFT slightly reduced the strength of association with dt only.

**Table 4:** Adjusted relation between K-CPQ scores and experience of dental caries.

Items	CPQ	CPQ Domain			
	Overall	Oral Symptoms	Functional Limitations	Emotional Well-being	Social Well-being
	partial r ^a^	partial r ^a^	partial r ^a^	partial r ^a^	partial r ^a^
CPQ_8–10_ (n = 355)					
dt	**0.153**	0.090	**0.124**	**0.233**	0.039
ft	-0.035	-0.026	-0.046	-0.001	-0.034
dft	0.044	0.021	0.022	**0.113**	-0.010
DT	-0.022	0.006	-0.018	-0.013	-0.041
FT	0.076	0.009	0.068	0.083	0.075
DMFT	0.059	0.011	0.053	0.069	0.050
dt+DT	**0.133**	0.084	**0.108**	**0.210**	0.023
dt+DMFT	**0.152**	0.076	**0.127**	**0.219**	0.059
CPQ_11–14_ (n = 446)					
DT	-0.066	-0.052	-0.035	-0.044	-0.072
MT	-0.039	-0.064	-0.029	-0.021	-0.011
FT	**0.144**	**0.158**	0.090	**0.117**	0.079
DMFT	0.090	**0.107**	0.059	0.079	0.033

**Bold** type denotes statistical significance at *p*-value <0.05.

^a^ partial r denotes partial correlation coefficient adjusted for age, gender and school type by multivariable linear regression model.

In subgroup analyses according to the type of schools (Table 5), the associations of K-CPQ_8–10_ overall score, functional limitation and emotional well-being domain scores with dt, ft, dt+DT and dt+DMFT were higher in public school children than in the total participants. Especially, the associations of K-CPQ_8–10_ overall score and oral symptoms became significant with ft and dft, which were not significant in the total participants.

**Table 5:** School type stratified association of K-CPQ_8–10_ scores with experience of dental caries.

School type Items	CPQ_8–10_	CPQ_8–10_ Domain			
	Overall	Oral Symptoms	Functional Limitations	Emotional Well-being	Social Well-being
	partial r ^a^	partial r ^a^	partial r ^a^	partial r ^a^	partial r ^a^
National (n = 118)					
dt	0.030	0.061	0.040	0.038	-0.065
ft	**0.188**	**0.193**	0.139	0.130	0.105
dft	**0.186**	**0.200**	0.143	0.134	0.079
DT	0.053	0.036	0.055	0.048	0.023
FT	0.024	-0.003	-0.060	0.060	0.072
DMFT	0.046	0.015	-0.023	0.074	0.071
dt + DT	0.051	0.065	0.059	0.054	-0.037
dt + DMFT	0.050	0.043	0.004	0.074	0.018
Public (n = 237)					
dt	**0.168**	0.093	**0.133**	**0.277**	0.045
ft	**-0.127**	0.193	-0.113	-0.073	-0.081
dft	-0.017	-0.053	-0.024	0.087	-0.044
DT	-0.046	-0.017	-0.037	-0.027	-0.057
FT	0.092	0.008	0.109	0.090	0.078
DMFT	0.069	0.001	0.089	0.074	0.052
dt + DT	**0.147**	0.083	0.116	**0.253**	0.029
dt + DMFT	**0.176**	0.077	**0.158**	**0.268**	0.065

**Bold** type denotes statistical significance at *p*-value <0.05.

^a^ partial r denotes partial correlation coefficient adjusted for age, gender and school type by multivariable linear regression model.

### Association between dental caries and K-CPQ_11–14_

The descriptive statistics showed that the K-CPQ_11–14_ overall score ranged from 0 to 74, with a mean of 8.49 and a standard deviation of 9.89. There were 39 children (8.7% of the total number of participants) with floor effect, while none of the children in the study had a ceiling effect. When the responses “often” or “very often” are counted for each child, the percentages of those reporting oral symptoms, functional limitations, emotional well-being and social well-being were 17.5%, 13.2%, 6.1%, and 5.4%, respectively.

In regards to objective oral health status (Table 4), the K-CPQ_11–14_ overall score had a positive correlation with the filled permanent teeth index [FT] (partial *r* = 0.14, *P* = 0.002). The score of FT was associated with only two domain scores: oral symptoms (partial *r* = 0.16, *P* = 0.001) and emotional well-being (partial *r* = 0.12, *P* = 0.014). Moreover, permanent dental caries experience (DMFT) was only positively associated with the oral symptoms score (partial *r* = 0.11, *P* = 0.025). However, the DMFT was not significantly associated with the K-CPQ_11–14_ overall score.

In the stratified analysis according to the type of school the child attended (Table 6), the associations of the K-CPQ_11–14_ overall score and the oral symptoms and emotional well-being domain scores with FT and DMFT were higher in children who attended public schools than in all of the total participants (partial *r* of overall scores = 0.18 versus 0.14).

**Table 6:** School type stratified association of K-CPQ_11–14_ scores with experience of dental caries.

School type Items	CPQ_11–14_	CPQ_11–14_ Domain			
	Overall	Oral Symptoms	Functional Limitations	Emotional Well-being	Social Well-being
	partial r ^a^	partial r ^a^	partial r ^a^	partial r ^a^	partial r ^a^
National (n = 139)					
DT	-0.107	-0.119	-0.075	-0.067	-0.093
MT	.	.	.	.	.
FT	0.104	0.087	-0.037	**0.177**	0.070
DMFT	0.012	-0.009	-0.077	0.089	-0.005
Public (n = 307)					
DT	-0.072	-0.040	-0.045	-0.045	-0.085
MT	-0.032	-0.064	-0.019	-0.017	-0.004
FT	**0.182**	**0.199**	**0.149**	**0.127**	0.093
DMFT	**0.128**	**0.155**	0.112	0.093	0.044

Bold type denotes statistical significance at *P*-value < 0.05.

^a^ partial r denotes partial correlation coefficient adjusted for age, gender and school type by multivariable linear regression model.

## Discussion

Two types of CPQ were developed to measure the OHRQOL among children between the ages of 8 and 10 years (CPQ_8–10_) and between the ages of 11 and 14 years (CPQ_11–14_) in Canada [11,12] and widely used in many English speaking countries (United Kingdom, New Zealand, Australia) and other countries (Mexico, Saudi Arabia, Denmark, Brazil, China). Hence, the CPQ was selected as the tool evaluating the OHRQOL among Korean children.

Cross-cultural adaptation of questionnaires of CPQ is necessary in order to make a viable information through the instrument developed in other languages and countries. The adaptation of such instruments for international research, however, poses a number of difficulties. First, there is the issue of language and cultural differences and thus the need to develop culturally equivalent measures [34]. Second, it is imperative that the psychometric properties of the instrument be assessed when employed in different settings as they should exhibit consistent findings by different researchers in different settings [21]. In our study, the backward translated English version was nearly the same as the original English version. Therefore, the CPQ is applicable to Korean children’s culture.

Our results showed that the intraclass correlation coefficients (ICCs) for K-CPQ_8–10_ were acceptable, which are suggesting substantial agreement with the previous study [35]. The ICCs of the test-retest reliability for the four domains in the Korean version (0.22–0.79) were similar to those in the Canada version (0.16–0.89) [12], but lower than Brazil version (0.85–0.94) [15]. Hence, the scores of test-retest reliability for K-CPQ_8–10_ overall and four domains scores were acceptable, except for the social well-being domains. Social functioning and experiences might be more likely to show variability over time than the physical and emotional effects of oral and orofacial conditions, especially for young children. The intraclass correlation coefficients (ICCs) for the K-CPQ_11–14_ were acceptable, which meets the guidelines of practical agreement [35]. The ICCs of the test-retest reliability for the four domains in the Korean version (0.67–0.88) were similar to those in the Canadian version (0.64–0.86) [11], but lower than the Brazilian version (0.84–0.95). Hence, the scores of test-retest reliability for the K-CPQ_11–14_ overall and the four domains were acceptable.

In terms of internal consistency, Cronbach’s alphas of K-CPQ_8–10_ domain questionnaires (0.57–0.77) were similar to the values obtained from the Canada version (0.63–0.78) [12] and Denmark version (0.57–0.78) [14]. However, it is lower than the values obtained from Australia version (0.65–0.88) [13] and Brazil version (0.67–0.92) [15]. It might be explained by the construct of 25 items. The questionnaires contain some items related to orofacial deformity such as cleft lip/cleft palate, which is rare in the general population. The idea about the change of items should be issued for developing more powerful instrument for the general population. Additionally, the volume of questionnaires was quite large for young children to fill out all 25 items. A short form of the CPQ_8–10_ could be useful to apply to a large population. The Cronbach’s alphas of the K-CPQ_11–14_ domain questionnaires (0.64–0.90) were similar to the values obtained from the Australian version (0.68–0.91) [13] and the Canadian version (0.64–0.86) [11]. However, this value was lower than the values obtained from the Brazilian version [15] and the Italian version except for the K-CPQ_11–14_ overall and the emotional well-being domain [26]. Some questionnaires contain items related to orofacial deformity such as cleft lip/cleft palate, which is rare in the general population. Some items should be reconsidered and altered in order to develop a more powerful instrument for the general population. Additionally, the questionnaire was quite long for young children to complete. Development of shorter forms of the K-CPQs could be useful when considering a large population.

The evidence that CPQ is associated with dental caries in the general population is not still clarified yet. The sample of non-association studies may be unsuitable since they may have discriminated against children with low caries levels. For this reason, if this measure is to be used in similar children with low levels of caries experience, a sufficient sample size will be required. Here, we estimated the sufficient sample size for this study by using the results of the pilot study. Moreover, most of the previous studies that used the CPQ assessed crude associations which were controversial by using the correlation coefficient and the mean comparison. Thus, the results of this study using the K-CPQ were adjusted for age, gender and school type by using linear regression models in order for the OHRQOL to be associated with socio- and demo-economic factors such as age, gender and school type. Overall, our results showed that the CPQ is independently associated with dental caries experience in school children after controlling for confounders. This suggests that the CPQ could be available to the general population to provide essential information for assessing OHRQOL among children.

Overall scores of K-CPQ_8–10_ had positive correlation with dental caries status. Especially, functional limitation and emotional well-being domains were positively associated with the dental caries status such as the prevalence and untreated dental caries in deciduous teeth after controlling for age, gender and schools. These could be addressed by the phenomena that deciduous untreated dental caries among children 8 to 10 could progress to become painful and stressful. Although K-CPQ_8–10_ overall score was also associated with objective oral health status including caries experience on deciduous/permanent teeth, DT and DMFT slightly attenuated the strength of association with dt only. It is considered that dental caries on permanent teeth may not be so severe for children aged 8 to 10 years to influence their OHRQOL.

Also, the overall scores of the K-CPQ_11–14_ had a positive correlation with both the FT and DMFT. The oral symptoms and emotional well-being domains were positively associated with the FT after controlling for age, gender, and school type. These results could be addressed by the phenomena that these children may have a variety of dental conditions and has received dental treatment in mixed dentition. Children between the ages of 11 to 14, have a clear understanding of complex emotions. Thus, emotional well-being was influenced by their dental status.

Psychological well-being of adolescents has shown to play a mediating role in OHRQOL. The relatively high contribution of the psychosocial characteristics (such as sense of coherence and/or self-esteem) may reflect the combination of a variety of psychosocial characteristics in determining CPQ_11–14_ responses [36], [37]. The impact of clinical status on children’s experiences of their oral health is often indirect and mediated through a host of psychological and social factors. Actually, in adolescents in New Zealand, there was no direct relationship between caries experience and OHRQOL using structure equation modelling, although caries experience was associated with the psychological characteristics factor [38]. Further studies including psychosocial factors will be needed to clarify the impact of psychosocial factors on the link between dental caries and CPQ.

We used school type as a surrogate of socioeconomic status. Our results of school type subgroup analyses showed that K-CPQ_8–10_ was related to untreated deciduous caries only for those attending public school. Also, the K-CPQ_11–14_ was only related to caries experience in children that attended public schools, with the exception of an association with emotional well-being and FT. Although the K-CPQ_11–14_ overall score in the total sample was also associated with FT and DMFT, the association with caries experience slightly increased in public schools. We speculated that the K-CPQ may be more applicable for non-affluent children living in Korea. Additional studies including direct socioeconomic factors are needed in order to better clarify this presumed association.

Our study had two limitations. First, the participants in our study were from the school children sample whose schools were selected by using cluster sampling. Since all samples may not fully represent all Korean children, the selection bias could have occurred and the association may be distorted. Finally, we used the indirect daylight according to the WHO guideline for evaluating dental caries, which created problems of underestimating dental caries. Although this instrumental problem could induce non-differential bias, the association between dental caries and CPQ might be distorted toward the null. Further well designed prospective study including representative samples and dental chair with dental light will overcome these limitations. Notwithstanding these limitations, this study could be valid enough to meet the objective to evaluate the association between K-CPQ and dental caries among Korean children and our results provided the substantially important evidence on the oral health promotion for children.

Overall, the K-CPQ was independently associated with dental caries among Korean children aged 8–14. The K-CPQ could be a practical tool to evaluate the subjective oral health among Korean children aged 8 to 14. Moreover, our data suggests that the CPQ could be applied to provide essential information for assessing oral symptoms, functional limitation, emotional and social well-being in relation to oral health among general children.

## Supporting Information

S1 Appendix Child Perceptions Questionnaire among children between the ages of 8 and 10 years (CPQ_8–10_) data set. Child Perceptions Questionnaire among children between the ages of 11 and 14 years (CPQ_11–14_) data set. Variable list.
(XLS)

## References

[1] Slade GD, Spencer AJ (1994) Development and evaluation of the Oral Health Impact Profile. Community Dent Health 11: 3–11. 8193981

[2] Sheiham A (2005) Oral health, general health and quality of life. Bull World Health Organ 83: 644 16211151 PMC2626333

[3] Allison P, Locker D, Jokovic A, Slade G (1999) A cross-cultural study of oral health values. J Dent Res 78: 643–649. 10029462 10.1177/00220345990780020301

[4] Bae KH, Kim HD, Jung SH, Park DY, Kim JB, et al (2007) Validation of the Korean version of the oral health impact profile among the Korean elderly. Community Dent Oral Epidemiol 35: 73–79. 17244140 10.1111/j.1600-0528.2007.00331.x

[5] Kushnir D, Zusman SP, Robinson PG (2004) Validation of a Hebrew version of the Oral Health Impact Profile 14. J Public Health Dent 64: 71–75. 15180074 10.1111/j.1752-7325.2004.tb02730.x

[6] Larsson P, List T, Lundstrom I, Marcusson A, Ohrbach R (2004) Reliability and validity of a Swedish version of the Oral Health Impact Profile (OHIP-S). Acta Odontol Scand 62: 147–152. 15370634 10.1080/00016350410001496

[7] Robinson PG, Gibson B, Khan FA, Birnbaum W (2003) Validity of two oral health-related quality of life measures. Community Dent Oral Epidemiol 31: 90–99. 12641588 10.1034/j.1600-0528.2003.00051.x

[8] Slade GD (1998) Assessing change in quality of life using the Oral Health Impact Profile. Community Dent Oral Epidemiol 26: 52–61. 9511843 10.1111/j.1600-0528.1998.tb02084.x

[9] Gherunpong S, Tsakos G, Sheiham A (2004) Developing and evaluating an oral health-related quality of life index for children; the CHILD-OIDP. Community Dent Health 21: 161–169. 15228206

[10] Pahel BT, Rozier RG, Slade GD (2007) Parental perceptions of children’s oral health: the Early Childhood Oral Health Impact Scale (ECOHIS). Health Qual Life Outcomes 5: 6 17263880 10.1186/1477-7525-5-6 PMC1802739

[11] Jokovic A, Locker D, Stephens M, Kenny D, Tompson B, et al (2002) Validity and reliability of a questionnaire for measuring child oral-health-related quality of life. J Dent Res 81: 459–463. 12161456 10.1177/154405910208100705

[12] Jokovic A, Locker D, Tompson B, Guyatt G (2004) Questionnaire for measuring oral health-related quality of life in eight- to ten-year-old children. Pediatr Dent 26: 512–518. 15646914

[13] Do LG, Spencer AJ (2008) Evaluation of oral health-related quality of life questionnaires in a general child population. Community Dent Health 25: 205–210. 19149296

[14] Wogelius P, Gjorup H, Haubek D, Lopez R, Poulsen S (2009) Development of Danish version of child oral-health-related quality of life questionnaires (CPQ_8–10_ and CPQ_11–14_). BMC Oral Health 9: 11 10.1186/1472-6831-9-11 19383176 PMC2679003

[15] Barbosa TS, Tureli MC, Gaviao MB (2009) Validity and reliability of the Child Perceptions Questionnaires applied in Brazilian children. BMC Oral Health 9: 13 10.1186/1472-6831-9-13 19450254 PMC2696414

[16] Goursand D, Paiva SM, Zarzar PM, Ramos-Jorge ML, Cornacchia GM, et al (2008) Cross-cultural adaptation of the Child Perceptions Questionnaire 11–14 (CPQ_11–14_) for the Brazilian Portuguese language. Health Qual Life Outcomes 6: 2 10.1186/1477-7525-6-2 18194552 PMC2246108

[17] Torres CS, Paiva SM, Vale MP, Pordeus IA, Ramos-Jorge ML, et al (2009) Psychometric properties of the Brazilian version of the Child Perceptions Questionnaire (CPQ11–14)—short forms. Health Qual Life Outcomes 7: 43 10.1186/1477-7525-7-43 19445725 PMC2689176

[18] Aguilar-Diaz FC, Irigoyen-Camacho ME (2011) Validation of the CPQ_8–10_ESP in Mexican School children in urban areas. Med Oral Patol Oral Cir Bucal 16: e430–435. 20711140 10.4317/medoral.16.e430

[19] Marshman Z, Rodd H, Stern M, Mitchell C, Locker D, et al (2005) An evaluation of the Child Perceptions Questionnaire in the UK. Community Dent Health 22: 151–155. 16161878

[20] Foster Page LA, Thomson WM, Jokovic A, Locker D (2005) Validation of the Child Perceptions Questionnaire (CPQ_11–14_). J Dent Res 84: 649–652. 15972595 10.1177/154405910508400713

[21] McGrath C, Pang HN, Lo EC, King NM, Hagg U, et al (2008) Translation and evaluation of a Chinese version of the Child Oral Health-related Quality of Life measure. Int J Paediatr Dent 18: 267–274. 10.1111/j.1365-263X.2007.00877.x 18554335

[22] Kolawole KA, Otuyemi OD, Oluwadaisi AM (2011) Assessment of oral health-related quality of life in Nigerian children using the Child Perceptions Questionnaire (CPQ11–14). Eur J Paediatr Dent 12: 55–59. 21434737

[23] Gururatana O, Baker S, Robinson PG (2011) Psychometric properties of long and short forms of the Child Perceptions Questionnaire (CPQ_11–14_) in a Thai population. Community Dent Health 28: 232–237. 21916360

[24] Bekes K, John MT, Zyriax R, Schaller HG, Hirsch C (2012) The German version of the Child Perceptions Questionnaire (CPQ-G11–14): translation process, reliability, and validity in the general population. Clin Oral Investig 16: 165–171. 10.1007/s00784-010-0496-5 21210166

[25] Brown A, Al-Khayal Z (2006) Validity and reliability of the Arabic translation of the child oral-health-related quality of life questionnaire (CPQ11–14) in Saudi Arabia. Int J Paediatr Dent 16: 405–411. 17014538 10.1111/j.1365-263X.2006.00775.x

[26] Olivieri A, Ferro R, Benacchio L, Besostri A, Stellini E (2013) Validity of Italian version of the Child Perceptions Questionnaire (CPQ11–14). BMC Oral Health 13: 55–61. 10.1186/1472-6831-13-55 24131892 PMC3856540

[27] Oscarson N, Kallestal C, Lindholm L (2007) A pilot study of the use of oral health-related quality of life measures as an outcome for analysing the impact of caries disease among Swedish 19-year-olds. Caries Res 41: 85–92. 17284908 10.1159/000098040

[28] Barbosa TS, Leme MS, Castelo PM, Gaviao MB (2011) Evaluating oral health-related quality of life measure for children and preadolescents with temporomandibular disorder. Health Qual Life Outcomes 9: 32 10.1186/1477-7525-9-32 21569403 PMC3115836

[29] Costa A, Ferreira MC, Serra-Negra JM, Pordeus IA, Paiva SM (2011) Impact of wearing fixed orthodontic appliances on oral health-related quality of life among Brazilian children. J Orthod 38: 275–281. 10.1179/14653121141632 22156183

[30] Vargas-Ferreira F, Ardenghi TM (2011) Developmental enamel defects and their impact on child oral health-related quality of life. Braz Oral Res 25: 531–537. 22147234 10.1590/s1806-83242011000600010

[31] Bendo CB, Paiva SM, Torres CS, Oliveira AC, Goursand D, et al (2010) Association between treated/untreated traumatic dental injuries and impact on quality of life of Brazilian schoolchildren. Health Qual Life Outcomes 8: 114 10.1186/1477-7525-8-114 20920332 PMC2959098

[32] del Carmen Aguilar-Diaz F, Irigoyen-Camacho ME (2011) Validation of the CPQ_8–10_ESP in Mexican school children in urban areas. Med Oral Patol Oral Cir Bucal 16: e430–435. 20711140 10.4317/medoral.16.e430

[33] WHO, editor (1997) Oral Health Surveys: Basic Methods. 4th edition ed Geneva: World Health Organization.

[34] Lawrence HP (2001) Cross-cultural perceptions of oral health and oral-health-related quality of life. Community Dent Health 18: 207–208. 11789696

[35] Landis JR, Koch GG (1977) The measurement of observer agreement for categorical data. Biometrics 33: 159–174. 843571

[36] Foster Page LA, Thomson WM, Ukra A, Farella M (2013) Factors influencing adolescents’ oral health-related quality of life (OHRQoL). Int J Paediatr Dent 23: 415–423. 10.1111/ipd.12011 23171387

[37] Foster Page LA, Thomson WM (2012) Caries prevalence, severity, and 3-year increment, and their impact upon New Zealand adolescents’ oral-health-related quality of life. J Public Health Dent 72: 287–294. 10.1111/j.1752-7325.2012.00336.x 22506615

[38] Foster Page LA, Thomson WM, Ukra A, Baker SR (2013) Clinical status in adolescents: is its impact on oral health-related quality of life influenced by psychological characteristics? Eur J Oral Sci 121: 182–187. 10.1111/eos.12034 23659241

